# Déterminants du refus de la vaccination contre le papillomavirus humain chez les parents des enfants de 9 à 14 ans au sein des ménages d’Aliodan en 2024, Côte d’Ivoire: étude transversale quantitative

**DOI:** 10.11604/pamj.2025.51.62.46844

**Published:** 2025-07-03

**Authors:** Marie Noelle Ano Ama Kounangui, Daniel Ekra Kouadio, Marielle Ehilé Assamala, Gilbert Konan Loukou, Christian Akani Bangaman, Chantal Kouassi

**Affiliations:** 1 Service de Surveillance Epidémiologie, Département de Santé Publique et Spécialités, Université Félix Houphouët Boigny, Abidjan, Côte d'Ivoire,; 2 Service de Surveillance Epidémiologique et du Programme Elargi de Vaccination, Département Veille Sanitaire et Médecine Préventive, Institut National Hygiène Publique, Abidjan, Côte d'Ivoire,; 3 Service Néphro-Pédiatrie, Département Mère Enfant, Université Félix Houphouët Boigny, Abidjan, Côte d'Ivoire

**Keywords:** Déterminants, refus, *human papillomavirus*, Côte d’Ivoire, Determinants, refusal, human papillomavirus, Ivory Coast

## Abstract

**Introduction:**

l'étude réalisée dans le district sanitaire de Treichville dans les ménages du quartier d’Aliodan, situé dans l'aire de santé d'Anoumabo en Côte d'Ivoire, visait à identifier les déterminants de refus de la vaccination contre le virus du papillome humain (VPH) chez les parents d'adolescentes âgées de 9 à 14 ans.

**Méthodes:**

il s'agit d'une étude transversale de type analytique à collecte prospective. Une analyse univariée a été réalisée à l'aide des tests exacts KHI 2 et Fischer pour comparer des groupes de variables. Une régression logistique multivariée pas à pas descendante a été utilisée pour identifier les déterminants du refus à la valeur p=0,05.

**Résultats:**

au total, 181 répondants ont été interrogés. Avec 62,98% de femmes contre 37,02% d'hommes avec un sex-ratio de 1,7. L'âge moyen était de 41,43 ± 11,19. Le taux de refus de la vaccination a été constaté chez 22,10% des enquêtés contre 77,90% qui n'avaient pas refusé de faire vacciner leurs enfants. Les déterminants du refus des parents des adolescents étaient liés aux parents ayant affirmé avoir scolarisé leurs adolescentes (p=0,0003; ORaj.= 0,1932; IC95%= [0,0795; 0,4693]), à l'origine ethnique étrangère (p=0,0106; ORaj.= 3,1636; IC95%= [1,3079; 7,6523]), aux parents sans appartenance religieuse (p=0,0002; ORaj.= 8,4600); IC95%= [0,7944; 3,9284]), à la difficulté d'accès aux transports (p=0,0111; ORaj.= 0,0534; IC95%= [0,0056; 0,5120]). Les sources d'information issues de la télévision ou des réseaux sociaux (p=0,0106; ORaj.=0,1322; IC95% = [0,0280; 0,624]).

**Conclusion:**

les facteurs socioculturels et pratiques ont contribué au refus des parents du vaccin contre le virus du papillome humain (VPH). D'où la nécessité de renforcer la formation des professionnels et des leaders communautaires pour les campagnes de sensibilisation de masse.

## Abstract (en)

**Introduction:**

this study, conducted in Treichville health district in households of the district of Aliodan within the Anoumabo health area in Ivory Coast, aimed to identify the factors associated with parental refusal of the human papillomavirus (HPV) vaccination for girls aged 9 to 14 years.

**Methods:**

we conducted an analytical cross-sectional study with prospective data collection. A univariate analysis was performed using Chi-square and Fisher's exact tests to compare variable groups. A stepwise descendant multivariate logistic regression was used to identify determinants of refusal at a significance level of p = 0.05.

**Results:**

a total of 181 respondents were interviewed, comprising 62.98% women and 37.02% men, with a sex ratio of 1.7. The average age was 41.43 ± 11.19 years. The rate of vaccine refusal was 22.10%, compared to 77.90% who did not refuse to vaccinate their children. The identified determinants of parental refusal included: having daughters enrolled in school (p=0.0003; Adjusted OR= 0.1932; 95% CI= [0.0795; 0.4693]), foreign ethnic origin (p=0.0106; Adjusted OR= 3.1636; 95% CI= [1.3079; 7.6523]), parents with no religious affiliation (p=0.0002; Adjusted OR= 8.4600; 95% CI= [0.7944; 3.9284]), difficulty accessing transportation (p=0.0111; Adjusted OR= 0.0534; 95% CI= [0.0056; 0.5120]), information sources limited to television or social media (p=0.0106; Adjusted OR= 0.1322; 95% CI= [0.0280; 0.624]).

**Conclusion:**

sociocultural and practical factors contributed to parental refusal of the HPV vaccine. This highlights the need to strengthen training for healthcare professionals and community leaders for effective mass awareness campaigns.

## Introduction

La vaccination constitue le plus grand succès dans la prévention des maladies infectieuses [1]. En particulier, la vaccination des adolescentes apparaît comme l'intervention la plus efficace à long terme pour réduire le risque de développer un cancer du col de l'utérus. Grâce à l'immunité collective, une large couverture vaccinale anti-PVH a protégé les personnes non vaccinées [2].

Cependant, depuis le début de l'histoire des vaccins, elle fait l'objet de controverse qui jette le doute sur son bien-fondé [1]. Des lobbys anti-vaccinaux très actifs sur les réseaux sociaux, des rumeurs infondées, amplifiées par les progrès technologiques et les changements sociaux érodent la confiance des parents dans la vaccination [3], avec une réticence grandissante de la part de la communauté [4]. À l'échelle mondiale, la couverture de la première dose de vaccin contre le papillomavirus humain chez les filles est passée de 20% en 2022 à 27% en 2023, ce qui est loin de la cible de 90% fixée pour 2030 [5].

La Côte d'Ivoire n'est pas en reste. Le taux d'incidence du cancer du col de l'utérus était de 31,2 cas pour 100 000 femmes, avec environ 2 067 nouveaux cas et 1 417 décès, soit un taux de mortalité estimé à 22,8 pour 100 000 femmes en 2020 [2]. Face à cette situation, le pays a introduit la vaccination contre le papillomavirus en novembre 2019. Mais du fait de certaines rumeurs, cette vaccination a connu un démarrage très difficile dans tous les districts sanitaires. Cependant, grâce à la sensibilisation sur le cancer du col de l'utérus, aux témoignages et l'organisation d'activité d'intensification réalisés en 2021, la population a fini par adhérer à la vaccination contre le HPV, qui connait aujourd'hui une forte demande surtout dans les établissements scolaires [6] tandis qu'il existe encore des difficultés pour atteindre les cibles au niveau de la communauté.

Aliodan, quartier de l'aire sanitaire de Marcory Anoumabo dans le district sanitaire de Marcory Treichville en Côte d'Ivoire, n'est pas épargné. La couverture vaccinale est très faible malgré l'intensification des activités de sensibilisation, la gratuité et la disponibilité du vaccin en routine. Pourquoi cette faible couverture vaccinale? En Côte d'Ivoire, la vaccination contre la HVP a été étendue à tous les districts durant ces cinq dernières années. Les travaux déjà menés [2] à notre connaissance sur le refus de la vaccination contre le virus du papillome humain chez les parents dans un contexte de routine n'ont encore été rapportés. Les objectifs de cette étude étaient d'identifier et de décrire les obstacles à la vaccination contre le HPV, d'analyser et de déterminer les facteurs associés aux refus de la vaccination contre le HPV par les parents des enfants de 9 à 14 ans dans les ménages du quartier d'Aliodan de l’aire sanitaire Marcory d’Anumado dans un contexte de prévalence élevée du cancer du col de l'utérus et de diversité culturelle de la population. L'identification de ces déterminants est importante pour mettre en œuvre des stratégies de promotion et de communication parmi des sous-groupes cibles spécifiques et l'amélioration de la couverture vaccinale et afin de réduire la prévalence du cancer du col de l'utérus.

## Méthodes

**Cadre de l'étude:** le quartier Aliodan est situé dans le village d'Anoumabo dans la commune de Marcory qui est à moins de 10km du District Sanitaire de Treichville-Marcory et à 1,8km de l'Hôpital général de Marcory, hôpital de référence de la commune de Marcory où se concentrent de nombreuses activités économiques dans un cadre semi-urbain. Il a une superficie d'environ 1,2km^2^ avec une densité de 38 108 hab/km^2^ et une population estimée à plus de 47 447 habitants. La couverture en HPV1 et 2 était respectivement de 4% à 1% sur la couverture globale qui était de 99% et 16% dans le district en 2021 [7]. En 2022, elle était de 6% à 4% sur 118% à 48% pour la couverture globale [8]. En 2023, elle était de 40% à 4% sur une couverture globale de 42% à 10% [9].

**Population d'étude:** la population est composée des parents des enfants de 9 à 14 ans dans les ménages du quartier Aliodan de Marcory Anoumabo.

### Critères de sélection

***Critère d'inclusion:*** être le parent ou le tuteur légal d'une adolescente résidant dans le quartier d'Aliodan; avoir dans le ménage des filles de 9 à 14 ans; disposer d'un carnet de vaccination.

***Critère de non-inclusion:*** les enquêtés résidant dans la zone d'étude et qui refusent de prendre part à l'étude; les enquêtés qui n'avaient pas de traducteur lors de notre passage.

**Type et période d'étude**: nous avons mené une étude transversale analytique avec une approche quantitative à collecte prospective, elle s'est déroulée du 1^er^ octobre 2024 au 31 janvier 2025.

**Echantillonnage:** pour le choix du quartier Aliodan, la sélection a été faite par la méthode d’échantillonnage stratifié équilibré dans deux groupes de performance de couverture vaccinale HPV2 (bonne, faible) en tenant compte du rapport des trois dernières années, à savoir de 2021 à 2023. Nous avons recherché la moyenne qui était de 25%, nous avons tiré au sort toutes les aires sanitaires du district de Treichville Marcory ayant une couverture vaccinale < 25% et > 25%. Et nous avons surechantillonné dans la zone à faible couverture contrairement à la zone de bonne couverture. Sur la base de la recherche des déterminants du refus de la vaccination contre le HPV, nous avons considéré les aires sanitaires ayant de très faible couverture vaccinale. Le quartier Aliodan de l'aire sanitaire d'Anoumabo a été retenu sur la base de ce critère.

***Calcul de la taille de l'échantillon***: elle a été déterminée par la formule de Schwartz:


$$\text{n}=Z^{2}p\left(1-p\right)/m^{2}$$

z= 1,96 à un niveau de confiance de 95%; m=7%, q= 1 - p et p=0,5; n= 0,5(1-0,5)1,96^2^\0,07^2^= 196 (taille minimale de l'échantillon). Les non-répondants ont été estimés à 10% de la taille de l'échantillon; ce qui revient à 196 x 10/100= 19,6= 196 + 19,6= 215,6 environ 216. En somme, la taille (N) de nos échantillons est de 216 enquêtés.

***Gestion des biais:*** les ménages facilement accessibles ont été privilégiés par rapport aux autres pour minimiser l'impact de la distance sur le choix du parent. Les carnets de vaccination ont aussi été priorisés comme source de données. Ces deux actions sont de nature à amoindrir les biais de sélection et de mémoire qui peuvent être associés aux sources de données et aux répondants âgés de l'enquête. La qualité des données a été contrôlée à plusieurs niveaux pour assurer leur fiabilité. Pour certains items, les enquêteurs s'en tenaient aux seules affirmations des mères et pères sans autres sources de vérification, il s'agissait des aspects socio-économiques.

### Techniques et outils de collecte des données

***Sélection des concessions:*** nous nous sommes placés au centre du quartier Aliodan, repéré par son centre de santé. A partir d'une pièce de monnaie dont les faces pile égale gauche ou face égale droite lors du lancer en l'air, ont permis de faire le choix de la direction. Les concessions trouvées dans la direction tirée au sort ont été numérotées. Le numéro de la première concession à visiter était choisi par tirage au sort. Après avoir sélectionné la première concession, l'enquêteur sélectionnait les concessions suivantes, de proche en proche, et en commençant toujours par la concession dont la porte était la plus proche. Lorsque la direction était épuisée, l'enquêteur retournait chaque fois à droite jusqu'à épuiser toutes les directions.

***Sélection des ménages:*** dans chaque concession, l'enquêteur recensait d'abord les ménages. Ces derniers étaient numérotés sur une liste et un était tiré au sort.

***Sélection des personnes:*** au sein de chaque ménage, des numéros ont été attribués aux personnes répondant aux critères d'inclusion. Un numéro était tiré au sort et la personne correspondante était enquêtée.

### Collecte des données

***Technique de collecte:*** des entretiens individuels structurés en face-à-face ont été menés auprès des personnes à enquêter. Pour les réponses incomplètes sur le vaccin, nous avons eu recours au carnet de vaccination (nom du vaccin anti-HPV, dose…).

***Outil de collecte:*** un guide d'entretien a été confectionné en fonction des objectifs de l'étude avec trois rubriques et 56 questions.

### Variables de l'étude

***Dépendante***: refus ; non-consentement explicite ou implicite des parents des adolescentes. C'est une variable binaire à deux modalités: [0]= Non et [1]= Oui, répondue à la suite de la réponse à la question, avez-vous refusé la vaccination contre le HPV pour votre enfant?

***Indépendante:*** le profil sociodémographique et économique portait sur l'âge, le sexe, le statut matrimonial, l'instruction, l'existence d'activités génératrices de revenus (AGr), la profession, le secteur d'activité, le nombre d'enfants.

La connaissance des parents sur l'infection à HPV et sur le vaccin: sources d'information, manifestations cliniques et mode de transmission de la maladie, moyens de prévention, les raisons de non-vaccination.

La perception s'intéresse au ressenti par rapport à la maladie et à la qualité perçue de la vaccination contre le HPV.

Les facteurs institutionnels et organisationnels comme: distance du ménage au centre de santé, moyen de transport et l'accueil.

**Saisie et analyse des données:** après téléchargement sur Excel pour l'épuration, les analyses pour les données quantitatives et qualitatives discrétisées ont été importées sur Epi Info version 2007 pour différentes analyses. L'analyse des données comportait deux parties: une partie descriptive et une partie analytique. A propos des résultats descriptifs, les fréquences ont été calculées avec leurs intervalles de confiance à 95% pour les variables qualitatives. Le calcul de moyennes avec leur écart-type a été effectué pour les variables quantitatives.

Pour les résultats analytiques, le test de KHI 2 ou Exact de Fisher a été utilisé selon ses conditions d'application pour comparer les proportions. Une analyse univariée a permis de retenir les facteurs associés au seuil de p<0,20. Une régression logistique a été utilisée pour identifier les facteurs associés au refus de la vaccination anti-VPH. Une démarche pas à pas descendante a permis de retenir les variables associées au refus de la vaccination au seuil p< 5%. L'adéquation du modèle final a été vérifiée par le test Likelihood Ratio.

**Considérations éthiques:** un consentement éclairé écrit a été demandé à toutes les personnes participant à l'étude. L'anonymat des personnes enquêtées a été respecté. Les données recueillies ont été gardées de façon confidentielle. Seuls les responsables de l'étude ont eu accès aux données. Une lettre d'information a été adressée aux responsables de zone.

## Résultats

Au total, 181 parents de 9 à 14 ans ont été interrogés.

**Figure 1:**
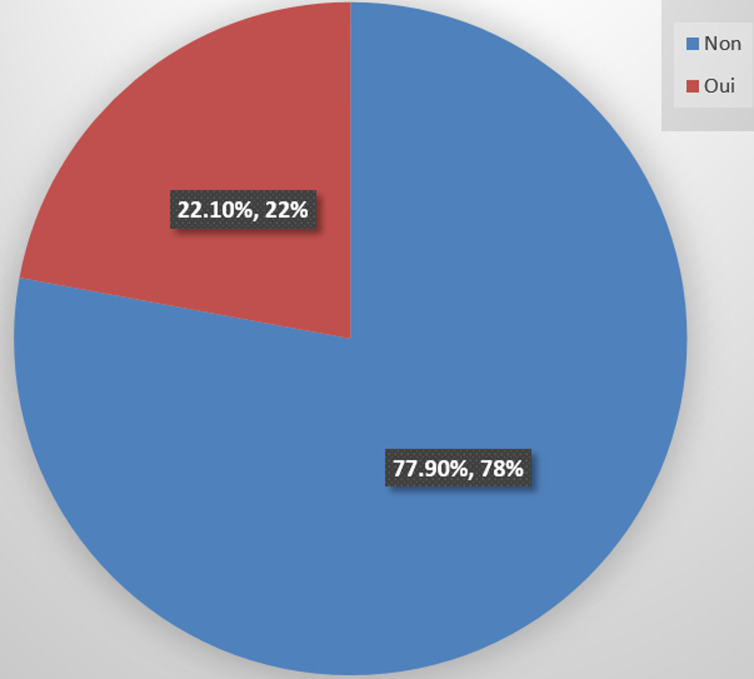
refus des parents des enfants de 9 à 14 ans des ménages d'Aliodan contre le vaccin anti-HPV, quartier de l’aire sanitaire de Marcory Anoumabo, district sanitaire de Treichville Marcory Côte d'Ivoire 2024

**Figure 2:**
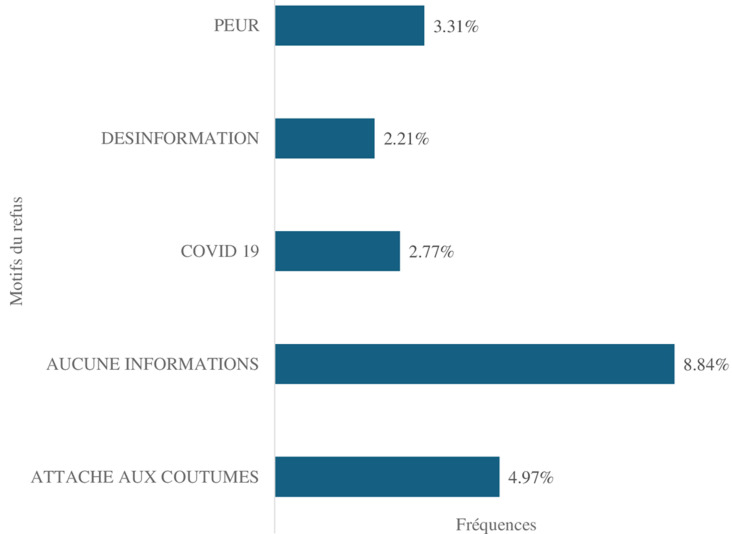
répartition des motifs de refus parmi les 40 enquêtés ayant refusé la vaccination anti-HPV dans les ménages d'Aliodan, quartier de l’aire sanitaire de Marcory Anoumabo, district sanitaire de Treichville Marcory Côte d'Ivoire 2024

**Description des caractéristiques socio-démographiques et professionnelles des enquêtés:** les femmes représentaient 62,98% des enquêtés contre 37,02% d'hommes avec un sex-ratio de 1,7. Des parents dont l'âge moyen était de 41,43 ± 11,19 avec le minimum à 23 ans et le maximum à 79 ans. Parmi ces enquêtés, la moitié était mariée dans 55,80% des cas et les célibataires représentaient 35,91% des cas. Plusieurs groupes ethniques ont été retrouvés dont les plus représentés étaient le groupe Akan, soit 37,22% des cas puis les étrangers avec 27,22% des cas et les Malinkés à 16,67% des cas. Groupe contrastant avec la religion, où les musulmans étaient représentés à 49,72% des cas suivis des chrétiens à 40,88% des cas. Concernant le niveau d'éducation, il y avait 67,40% des enquêtés qui étaient diplômés et 32,60% qui n'avaient aucun diplôme. Les parents ayant un niveau d'éducation élevé ont aussi un souci de l'éducation de leurs enfants. Ainsi, ils ont affirmé avoir des enfants scolarisés dans 86,74% des cas contre 13,26% des cas qui ne l'avaient pas fait. Nous avons recensé 35,95% de travailleurs indépendants suivis de 24,41% sans profession. Les fonctionnaires et employés du privé étaient respectivement de 17,68% et de 16,57% des cas. Aussi, le refus de la vaccination a été constaté chez 22,10% des enquêtés contre 77,90% qui n'avaient pas refusé de vacciner leurs enfants (Figure 1). La prise de décision était faite par la mère dans 81,77% des cas contre 17,13% des cas par le père et 1,10% des décisions qui étaient prises par les deux parents. Les motifs de refus évoqués parmi les parents ayant refusé ont été dans 8,84% des cas la peur, 4,97% des cas le fait qu'ils soient attachés à la coutume, dans 3,31% des cas le fait qu'ils ne savent pas, dans 2,76% des cas à cause de la COVID-19 et dans 2,21% des cas la désinformation (Figure 2). Les sujets relatifs à la sexualité n'étaient pas abordés par les parents des enfants dans 48,52% des cas. Par ailleurs, 25,41% des enquêtés vivaient entre 501 et 1000 m du centre de santé versus 9,39% qui vivaient à plus de 1000m et un véhicule a été utilisé dans 14,36% des cas pour se rendre au centre de santé. Et le personnel soignant lors des visites des parents au centre de santé pour la vaccination des enfants, a été reconnu accueillant dans 91,71% contre 8,29% qui ne l'ont pas trouvé.

**Description des connaissances des parents des adolescentes sur la politique vaccinale en matière de HPV:** nous avons les sources d'information qui étaient diversifiées à savoir le centre de santé (37,57%), la télévision (33,70%), la famille (6,08%), la communauté (4,97%), du bouche à oreille (3,87%), le médecin traitant (2,76%), les réseaux sociaux (2,21%), au travail (1,66%) et 6,63% qui n'avaient aucune source d'information sur le vaccin contre le HPV. Aussi, les maladies causées par le HPV étaient connues par 43,09% des enquêtés contre 32,60% qui ne les connaissaient pas et 24,31 ignoraient l'existence du HPV. La majorité des enquêtés, soit 65,19% savaient quelle maladie le papillomavirus était responsable contre 34,81% qui ne le savaient pas. Le mode de transmission du HPV était connu par les 2/3 des enquêtés soit 67,96% contre 32,03% qui ne le savaient pas. Quant aux moyens de prévention, 68,51% des enquêtés les connaissaient contre 31,49% des enquêtés qui ne les connaissaient pas. Le vaccin contre le HPV a été cité pour la prévention contre le cancer par des enquêtés dans 59,67% des cas, 34,81% ont dit avoir des doutes et 5,52% réfutaient le vaccin contre le HPV comme prévention contre le cancer.

**Description des perceptions des parents des adolescentes sur la politique vaccinale en matière de HPV:** la dangerosité du vaccin a été reconnue par 20,44% des enquêtés contre 79,56% qui ne le trouvaient pas dangereux. Concernant la fiabilité, 53,98% de la moitié des enquêtés ont reconnu le vaccin fiable suivi de 18,75% qui ne sont pas encore convaincus totalement et 27,27% qui ne croient pas en la fiabilité du vaccin. Par ailleurs, l'influence de la coutume pour certains enquêtés a été reconnue comme incompatible pour 13,81% d'entre eux, puis 55,80% l'ont reconnue compatible avec leur coutume et 30,39% ne savent pas s'il était compatible avec leur coutume.

### Analyse statistique

**Analyse univariée**

***Caractéristiques socio-démographiques associées au refus de la vaccination contre le HPV par les parents des adolescents:*** le sexe (p=0,1578), la profession (p=0.1695), les parents ayant affirmé scolariser leurs adolescentes (p=0.0004), l'ethnie (P=0,0466), la croyance (p=0,0549), religion(p=0,0006), distance(p=0,049), moyen de transport (p=0,0090) étaient associés au refus de la vaccination contre le HPV (Tableau 1).

**Tableau 1:** caractéristiques socio-démographiques associées au refus de la vaccination contre le HPV par les parents des enfants de 9 à 14 ans des ménages d'Aliodan, quartier de l’aire sanitaire de Marcory Anoumabo, district sanitaire de Treichville Marcory Côte d'Ivoire 2024

caractéristiques socio-démographiques		Avez-vous refusé la vaccination contre le HPV de votre adolescent de 9-14 ans ?		
**Variables**	**-**	**Non**	**Oui**	**p-value**
**-**	**Sexe**	**-**	**-**	**-**
**-**	**Masculin**	56(39.72)	11(27.50)	0,1578
**-**	**Féï¿½minin**	85(60.28)	29(72.50)	**-**
**Tranche d'âge**	**-**	**-**	**-**	**-**
**-**	[23-30]	26 (18,44)	8 (20,00)	0,5519
**-**	[31-40]	47 (33,33)	14 (35,00)	
**-**	[41-50]	39 (27,66)	7 (17,50)	
**-**	[51-79]	29 (20,57)	11 (27,50)	
**-**	**Situation matrimoniale**	**-**		**-**
**-**	**Divorcés**	5(3.55)	2(5.00)	0.6104
**-**	**Célibataires**	54(38.30)	11(27.50)	
**-**	**Mariés**	76(53.90)	25(62.50)	
**-**	**Veufs**	6(4.26)	2(5.00)	
**Profession**	**-**	**-**	**-**	**-**
**-**	**Sans profession**	39(27.66)	15 (37.50)	0.1695
**-**	**Fonctionnaires**	28(19.86)	4 (10.00)	
**-**	**Travailleurs indépendants**	48(34.04)	17 (42.50)	
**-**	**Employés**	26(18.44)	4 (10.00)	
**Adolescente scolariséï¿½es**	-	-	-	-
**-**	**Non**	12(8.51 %	12(30.00)	0.00040
**-**	**Oui**	129(91.49)	28(70.00)	
**Ethnie**	-	-		
**-**	**We**	14(9.93)	3(7.69)	0,0466
**-**	**Akan**	58(41.13)	10(25.64)	
**-**	**Etrangers**	31(21.99)	18(46.15)	
**-**	**Krou**	15(10.64)	2(5.13)	
**-**	**Malinke**	23(16.31)	6(15.38)	
**Croyances**	-	-	-	-
**-**	**Non**	57(40.43)	23(57.50)	-
**-**	**Oui**	84(59.57)	17(42.50)	0.0549589676
**Religion**	-	-	-	-
**-**	**Sans religion**	7(4.96)	10(25.00)	0,0006
**-**	**Chrétiens**	77(54.61)	13(32.50)	
**-**	**Musulmans**	57(40.43)	17(42.50)	
**Distance**	-	-		
**-**	**Aucun**	12(8.51)	7(17.50)	0,0497
**-**	[100-200]	41(29.08)	8(20.00)	
**-**	[300-700]	56(39.72)	10(25.00)	-
**-**	[1000-7500]	32(22.70)	15(37.50)	-
**Moyen de transport utilisé pour se rendre au centre de santé**	**-**	**-**	**-**	**-**
**-**	**A pied**	108(76.60)	33(82.50)	0.0090
**-**	**Moto**	8(5.67)	6(15.00)	
**-**	**Voiture**	25(17.73)	1(2.50)	

***Données sur les connaissances et perceptions associées au refus de la vaccination contre le HPV par les parents des adolescents***

***Données de connaissance associées au refus de la vaccination:*** il existait un lien statistiquement significatif entre les sources d'informations (p=0,0041) et le refus de la vaccination contre le HPV (Tableau 2).

**Tableau 2:** données de connaissance associées au refus de la vaccination des parents des enfants de 9 à 14 ans des ménages d´Aliodan, quartier de l’aire sanitaire de Marcory Anoumabo, district sanitaire de Treichville Marcory Côte d'Ivoire 2024

Données sur les connaissances	Refus		
**Variables**	**Oui**	**Non**	**p-value**
Sources d'informations			
Aucune information	13(9.22)	2(5.00)	0,0041
Télévision et réseaux sociaux	59(41.84)	6(15.00)	
Médecin et centre de santé	50(35.46)	23(57.50)	
Famille et communauté	19(13.48)	9(22.50)	
**Connaissez-vous les maladies causées par le HPV?**			
Oui	64(45.39)	14(35.00)	0,2415
Non	77(54.61)	26(65.00)	
**Mode de transmission du virus**			
Je ne sais pas	98(69.50)	25(62.50)	-
Rapport sexuel	41(29.08)	13(32.50)	0.2674
Salive et sang	2(1.42)	2(5.00)	-

***Données de perception associées au refus de la vaccination:*** il existait un lien statistiquement significatif entre la perception de la population face au vaccin notamment sur des coutumes (p=0,0187), la compatibilité avec leurs religion (p=0,0549) et le refus de vaccination (Tableau 3).

**Tableau 3:** données de perception associées au refus de la vaccination des parents des enfants de 9 à 14 ans des ménages d'Aliodan, quartier de l’aire sanitaire de Marcory Anoumabo, district sanitaire de Treichville Marcory Côte d'Ivoire 2024

Refus			
**Variables**	**Non**	**Oui**	p-value
**Données sur la perception**			
**Que pensez-vous du vaccin contre le HPV?**			
Dangereux	10(7,09)	3(7,50)	0,1256
Prévention contre le cancer du col de l'utérus	2(1,42)	3(7,50)	
Rien	129(91,49)	34(85,00)	
**Influence des coutumes**			
Je ne sais pas	35(24,82)	20(50,00)	0,0187
Non	22(15,6)	3(7,50)	
Oui	84(59,57)	17(42,50)	
**Le vaccin contre le HPV est-il compatible avec votre religion?**			
Non	57(40,43)	23(57,50)	0,0549589676
Oui	84(59,57)	17(42,50)	

**Analyse multivariée:** il existait un lien statistiquement significatif entre les parents ayant affirmés scolarisés leurs adolescentes (p=0,0003; ORaj= 0.1932; IC95%= [0.0795; 0.4693]), l'ethnie étrangère (p=0,0106; ORaj= 3.1636; IC95%= [1.3079; 7.6523]), les enquêtés se rendant au centre de santé en voiture (p=0,0111; ORaj= 0.0534; IC95%= [0.0056; 0.5120]), les sans religions (p=0,0002; ORaj= 8.4600; IC95%= [0.7944; 3.9284]) et le refus de vaccination. Aussi, entre la connaissance des conséquences du HPV (p=0,0089; ORaj= 0.3838; IC95%= [0.1872; 0.7869]), la télévision et les médias comme source d'information (p=0,0106; ORaj= 0.1322 ; IC95%= [0.0280; 0.624) et le refus de vaccination; entre la compatibilité du vaccin contre le HPV et leurs religions (p=0) et le refus de vaccination. Enfin entre la compatibilité du vaccin contre le HPV et leurs religions (p=0,0573; ORaj= 0.5016; IC95%= [0.2463; 1.02160573; ORaj= 0.5016; IC95%= [0.2463; 1.0216])) et le refus de vaccination (Tableau 4).

**Tableau 4:** déterminants du refus de la vaccination contre le HPV par les parents des enfants de 9 à 14 ans les ménages d’Aliodan, quartier de l’aire sanitaire de Marcory Anoumabo, district sanitaire de Treichville Marcory Côte d'Ivoire 2024

Refus			
**Variables**	**ORaj**,	**IC 95%**	**p-Value**
**Caractéristiques socio-démographiques**			
**Votre adolescent est-il scolarisé?**			
Oui	0,1932	[0,0795-0,4693]	0,0003
Non	**Réf;**		
Ethnies			
Akan **Réf;**			
Etrangère	3,1636	[1,3079-7,6523]	0,0106
Krou	1,0000	[0,3127-3,1975]	1,0000
Malinke	1,9333	[0,6518-5,7344]	0,2347
**Transport utilisé pour se rendre au centre de santé**			
**Moto Réf;**			
Pied	0,4074	[0,1319-1,2587]	0,1187
Voiture	0,0534	[0,0056-0,5120]	0,0111
**Religion**			
Chrétien **Réf;**			
Musulmans	1,7665	[2,7312-26,2053]	0,1629
Sans religion	8,4600	[0,7944-3,9284]	0,0002
**Données sur les connaissances**			
**HPV est responsable**			
Cancer du col de l'utérus	**Réf**,		0,0089
Je ne sais rien	0,3838	[0,1872-0,7869]	
**Source d'informations**			
Aucune **Réf;**			
Centre de santé	1,2723	[0,4046-4,0009]	0,6803
Communauté	1,0636	[0,2927-3,8657]	0,9253
Télévision et médias	0,1322	[0,0280-0,624]	0,0106
**Données sur la perception**			
**Le vaccin contre le HPV est-il compatible avec votre religion?**			
Non	**Réf**,		0,0573
Oui	0,5016	[0,2463-1,0216]	

## Discussion

Cette étude avait pour but de déterminer les facteurs associés aux refus de la vaccination contre le HPV par les parents des enfants de 9 à 14 ans dans les ménages de l'aire sanitaire d'Aliodan en Côte d'Ivoire à travers une étude analytique à collecte prospective. Elle a permis de mettre en lumière un taux de refus à 22,10% supérieur à celui retrouvé à Gao au Mali en 2021 qui était de 9,11% [3]. Ce taux de refus vaccinal élevé dans notre étude s'expliquait pour 8,84% des cas par la peur « effets indésirables », 4,97% des cas, du fait qu'ils soient attachés à la coutume, dans 3,31% des cas par le manque d'informations, dans 2,76% des cas à cause de la COVID-19 et dans 2,21% des cas par la désinformation. Par ailleurs, plusieurs facteurs significativement associés au refus de la vaccination contre le HPV à savoir les parents ayant affirmé scolariser leurs adolescentes, l'ethnie étrangère, l'influence des croyances, les parents sans religion, la voiture comme mode de transport pour se rendre au centre de santé ont été retrouvés. Aussi, y ont contribué, la méconnaissance des conséquences du HPV ainsi que la télévision et les médias comme sources d'information.

**Description des facteurs socio-démographiques des enquêtés:** en effet, il a été retrouvé un lien statistiquement significatif entre les parents ayant affirmé scolariser leurs adolescentes et ceux l'ayant pas fait. Cela pourrait s’expliquer par le fait que ces parents jugent leurs filles "trop jeunes" pour un vaccin lié à une infection sexuellement transmissible (IST), retardant la vaccination jusqu’à l’adolescence avancée. Aussi dans des contextes conservateurs, aborder la vaccination HPV reviendrait à évoquer la sexualité, ce qui est tabou pour des enfants de 9-14 ans [10] et faciliter ainsi l'acceptation du vaccin. Dainguy *et al*. [2], ont montré que l'éducation est un déterminant clé des comportements de santé. Une meilleure éducation permet non seulement une meilleure compréhension des bénéfices des vaccins, mais aussi une plus grande capacité à accéder à des informations fiables sur la santé [11].

Par ailleurs, les observations faites par N'diaye, qui souligne que l'ethnie joue également un rôle important dans le refus de la vaccination contre le HPV [12], ont été retrouvées dans nos résultats. Cette relation met en lumière le rôle des traditions culturelles et des perceptions sociales au sein des différentes communautés ethniques. Les membres de certaines ethnies peuvent être moins enclins à accepter la vaccination en raison de croyances culturelles ou de la perception que la vaccination est contraire à leurs pratiques traditionnelles. Par exemple, dans certaines communautés Yacouba ou Dioula, il existe une méfiance envers les interventions médicales extérieures considérées. Les différences culturelles et ethniques sont souvent des obstacles à l'acceptation de la vaccination.

De même, les croyances personnelles des parents jouent un rôle important dans leur décision de faire vacciner ou non leurs enfants. La p-value de 0,0549 montre que les croyances, notamment celles liées à la peur des effets secondaires ou à l'impact sur la fertilité, peuvent influencer la décision de vaccination. Certains parents croient à tort que la vaccination contre le HPV rend stérile [4], la peur des effets indésirables ou des rumeurs liées à la vaccination (par exemple, des liens supposés avec l'autisme ou l'infertilité) contribue largement à la réticence envers les vaccins des filles sexuellement actives prématurément, un mythe largement répandu dans de nombreuses communautés. A cela, s'ajoute le facteur religieux qui est aussi un déterminant important. Certaines croyances religieuses peuvent interdire ou limiter la vaccination, particulièrement dans des communautés conservatrices ou pratiquant des religions ayant des doctrines strictes sur les soins de santé. Par exemple, certains groupes protestants ou musulmans peuvent être réticents à accepter le vaccin contre le HPV en raison des croyances liées à la pureté sexuelle et à la préservation de la virginité. Cette tendance est observée dans des études réalisées au Nigeria et en Indonésie, où les préoccupations religieuses ont été un obstacle majeur à la vaccination contre le HPV [12].

La distance géographique entre le domicile des familles et le centre de vaccination est également un facteur significatif. Impliquant que, les familles vivant loin des établissements de santé sont moins susceptibles de faire vacciner leurs filles.

En effet, quoique gratuite dans les programmes nationaux, la vaccination peut avoir des coûts supplémentaires onéreux pour les familles pauvres, il s'agit par exemple des frais de transport. Ainsi, l'accès limité aux centres de santé, combiné à des infrastructures de transport insuffisantes, réduit l'adhésion au programme de vaccination. Ce phénomène a été observé dans des études sur la vaccination en zone rurale en Afrique et en Asie, où les communautés éloignées des centres de santé sont confrontées à des obstacles géographiques importants [13]. Salah suggérait que des programmes de vaccination mobiles ou des initiatives locales pourraient améliorer l'accès aux vaccins pour ces populations. Eu égard la distance, le mode de transport (p-value de 0,0090) est un autre facteur déterminant. Les familles disposant d'un transport facile et rapide vers les centres de santé sont plus susceptibles de faire vacciner leurs filles. En revanche, ceux qui n'ont pas accès à des moyens de transport fiables sont plus enclins à refuser la vaccination en raison des difficultés d'accès aux services de santé. Aussi, Djimet, sur les barrières à la vaccination, a révélé que l'absence de transport était un obstacle majeur à la vaccination dans de nombreuses régions rurales des pays en développement [14].

**Déterminants du refus de la vaccination contre le HPV:** les perceptions et les connaissances des parents jouent un rôle central dans la décision d'accepter ou de refuser la vaccination contre le HPV pour leurs adolescentes. En effet, une p-value de 0,0041 entre les sources d'information et le refus de la vaccination montre l'importance de l'accès à des informations fiables et claires.

Selon l'OMS 2016, en raison du développement de l'accès à internet et des réseaux sociaux, l'information, faits et rumeurs, célébrations et peurs peuvent se diffuser plus rapidement qu'avant. Les événements ou perceptions du public dans d'autres pays, voire d'autres continents, peuvent avoir un impact sur la compréhension des populations et la confiance dans les vaccins [10]. Ainsi, les perceptions, influencées par les sources d'information, peuvent avoir un impact profond sur la décision des parents de faire vacciner ou non leurs filles contre le HPV. Soulignant l'importance de la manière dont les informations circulent dans la communauté et les familles. Les parents qui reçoivent des informations provenant de sources fiables (professionnels de santé, campagnes de santé publique) sont généralement plus enclins à accepter la vaccination. En revanche, ceux qui s'appuient sur des sources douteuses, comme des rumeurs, des amis ou des médias sociaux non vérifiés, sont plus susceptibles de douter de l'efficacité et de la sécurité du vaccin. L'étude de Dainguy *et al*. a révélé que les informations erronées véhiculées par des canaux non professionnels (tels que les réseaux sociaux, les blogs ou les médias populaires) peuvent grandement influencer les perceptions des parents et susciter des réticences à la vaccination. Par exemple, des rumeurs de liens fictifs entre le vaccin contre le HPV et l'autisme ou la stérilité ont alimenté la méfiance envers la vaccination dans plusieurs pays, y compris en Afrique et en Europe [2].

Dans le contexte de l'aire sanitaire d'Anoumabo, les sources d'information primaires des parents sont souvent les médias sociaux ou les rumeurs dans leur communauté. Un exemple concret d'influence négative provient des groupes de discussion informels où des informations fausses ou mal interprétées sont largement partagées. Ainsi, la désinformation peut entraîner une perception erronée du vaccin et de ses effets secondaires supposés, poussant certains parents à refuser la vaccination de leurs filles. Par exemple, en septembre 2011, un débat s'était engagé aux États-Unis sur la vaccination des jeunes filles par le vaccin HPV, et suite aux déclarations d'une députée qui accusait ce vaccin d'être responsable des réactions dangereuses et même d'avoir provoqué un retard mental chez une fille de douze ans. La revue *Nature* avait fait remarquer la gravité de ces incitations au refus vaccinal [15].

D'où le rôle des professionnels de santé dans la diffusion des messages de santé. D'autre part, les professionnels de santé jouent un rôle primordial dans la formation des perceptions des parents concernant le vaccin contre le HPV. Selon l'OMS, les informations données par des médecins ou des infirmières sont souvent perçues comme plus crédibles et fiables. Les parents qui ont eu des interactions positives avec des professionnels de santé, qui ont reçu des explications claires sur la sécurité et l'efficacité du vaccin, sont plus susceptibles de vacciner leurs enfants [16]. Cependant, dans certains contextes, notamment en Côte d'Ivoire, les professionnels de santé peuvent ne pas toujours disposer des ressources nécessaires ou des formations suffisantes pour aborder efficacement les préoccupations des parents. Une étude en Côte d'Ivoire a montré que les jeunes adultes et parents sont plus enclins à écouter les médecins qui peuvent répondre à leurs préoccupations de manière détaillée et rassurante [17].

Cela met en lumière l'importance des formations continues pour les professionnels de santé afin de s'assurer qu'ils possèdent des informations actualisées sur le HPV et la vaccination, et qu'ils soient capables de répondre aux préoccupations des parents de manière compréhensible et respectueuse.

Cette situation pourrait créer la méfiance envers les autorités sanitaires constituant ainsi un facteur majeur qui influence les perceptions des parents. Les campagnes de vaccination, bien qu'ayant l'intention de fournir des informations objectives et basées sur des données scientifiques, peuvent parfois échouer à atteindre les populations locales si elles ne sont pas adaptées aux contextes sociaux et culturels.

Un exemple de ce phénomène a été observé dans une étude menée en Côte d'Ivoire où, dans certaines zones rurales, les campagnes de vaccination ont été mal accueillies car, les informations transmises étaient perçues comme imposées par des acteurs externes (ONG internationales ou gouvernement) sans suffisamment de dialogue local préalable. Ce type de méfiance institutionnelle pousse les parents à rechercher des informations alternatives, souvent biaisées, augmentant ainsi leur réticence à vacciner leurs enfants [14].

Dans un contexte plus large, selon l'OMS, dans les pays où la confiance dans les autorités de santé publique est faible, l'adhésion à la vaccination, y compris contre le HPV, est considérablement réduite [10]. D'autres facteurs tels que les croyances culturelles et religieuses ont également un impact profond sur les perceptions des parents concernant la vaccination contre le HPV. Dans certaines communautés ivoiriennes, par exemple, des croyances liées à la pureté sexuelle et à la préservation de la virginité peuvent jouer un rôle crucial. Certains parents croient que la vaccination contre le HPV pourrait inciter leurs filles à adopter un comportement sexuel prématuré, bien que le vaccin soit destiné à prévenir les cancers liés au HPV et non à encourager l'activité sexuelle [18].

Dans des études menées dans divers pays africains, les préoccupations relatives à la virginité et à la moralité sexuelle sont des arguments fréquemment cités contre la vaccination. Les messages de prévention sur la vaccination contre le HPV doivent donc être adaptés à ces perceptions culturelles afin de contourner ce type de résistance [19].

Les perceptions des effets secondaires du vaccin sont également un facteur clé. Beaucoup de parents craignent des réactions indésirables graves, comme des douleurs localisées, de la fièvre ou des effets plus graves, bien que ces effets soient rares et souvent modérés. Le manque de compréhension des effets secondaires peut pousser certains parents à refuser la vaccination par peur des risques.

Ainsi, les perceptions erronées des effets secondaires, souvent véhiculées par des sources non vérifiées (amis, famille, médias sociaux), sont des barrières majeures à la vaccination. Les campagnes de sensibilisation devraient donc mettre l'accent sur la sécurité des vaccins, basées sur des données scientifiques fiables, pour dissiper ces préoccupations [14].

## Conclusion

L'étude menée dans le quartier Aliodan, dans l'aire sanitaire de Marcory d'Anoumabo en Côte d'Ivoire, a mis en lumière les principaux déterminants du refus de la vaccination contre le HPV les parents ayant affirmés scolariser leurs filles, les croyances culturelles et religieuses, les défis logistiques liés à l'accès aux centres de vaccination, le nombre d'enfants en bas âge et l'exposition aux réseaux sociaux. Pour améliorer l'acceptation de la vaccination contre le HPV, il est essentiel de renforcer l'éducation des jeunes filles, de mobiliser les professionnels de santé comme sources crédibles d'information et de cibler des stratégies de communication adaptées aux contextes culturels et religieux locaux.

### Etat des connaissances sur le sujet

- Le phénomène du refus de la vaccination est étudié dans le monde et son cadre conceptuel et consensuel a été défini par le SAGE mise en place par l'OMS;
- C'est l'une des 10 menaces pour la santé mondiale;
- Il n'est pas assez étudié dans les pays en développement, avec très peu d'études en Afrique où les maladies évitables par la vaccination font des centaines de victimes chaque année, particulièrement chez les jeunes filles exposées au risque de maladies à transmission sexuelle voire aux affections dues au HPV.

### Contribution de notre étude à la connaissance

- Notre étude apporte pour la première fois un taux de refus vaccinal de 22,10% dans un district de la ville d'Abidjan;
- Il décrit la diversité culturelle de la population enquêtée et la prise de décision vaccinale faite par les mères des adolescents;
- Les résultats de notre étude suggèrent que les parents ayant un niveau supérieur sont en train d'une réticence culturelle constituant un obstacle à la vaccination de leurs adolescents.

## Remerciements

Nous remercions tout le personnel du district sanitaire de Treichville Marcory et les communautés d'Aliodan.
